# Authors’ reply

**Published:** 2010

**Authors:** S. Priyadarshini, J. S. Ashadevi, V. Nagarjun, K. S. Prasanna

**Affiliations:** *Government Ayurveda Medical College, Mysore, Karnataka, India. E-mail: shantala301@gmail.com*; 1 *Yuvaraja’s College, University of Mysore, Mysore, India*; 2 *Uppsala University, Sweden*; 3 *Maharani’s Science College for Women, Mysore, India*

Sir,

This is in response to the letter from Lakhotia S related to our work published as a preliminary report.[1,2] Professor Lakhotia is right in pointing out the need for more studies on rasayana effects in animal models. We also appreciate his concerns and questions related to our article.

It is important to understand that rasayana relates to *rasa*, which not only has organism-specificity but it is also dependent on other significant factors. According to Sushruta Samhita (*sarvabhuta chinta sharera Su. Sm. Sh chapter 1/1–3*), all living organisms have certain features in common, and others that distinguish them.

Our earliest research started in June 2007, when we began a series of tests on four commercial formulations of human-optimized rasayanas on small numbers of flies. Some of these showed marginal changes in longevity, but none were striking, nor what we expected for a “*rasayana*-effect.” On the contrary, we observed significant adverse effects including lethality. This suggested that the paste-like dosage form of *rasayana formulations prescribed for humans* was adversely affecting the feeding process. This led us to consider developing “*Drosophila*-friendly” rasayana formulations. Basic principles of Ayurveda especially of dravyaguna shastra provide the way for developing such preparations. Rasayana in liquid dosage forms would provide an easy way to alter the concentration in the feed and to arrive at precisions stated in our paper as “1 drop of *Drosophila* rasayana food supplement.”

A considerable amount of innovation went into these developments and we wanted to protect the intellectual property. We thank *J-AIM* editors for agreeing to a black box approach in our reporting the experiments, and to our withholding details of its formulation. However, we confirm that the details of the formulation including chemical standardization data will be made available to J-AIM once patent formalities are completed.

The extensive introduction was included to communicate fundamental understanding of basic principles to suit the diverse backgrounds of the multidisciplinary readership of *J-AIM*. Both reported longevity experiments were made on *D. melanogaster* Oregon-K strain obtained from the *Drosophila* Stock Center, Department of Zoology, Manasagangothri, University of Mysore. Our research team included both experienced *Drosophila* scientists and Ayurveda experts. We followed standard experimental protocols and do not understand the grounds on which Lakhotia claims that the study was poorly executed. However, we do appreciate his comments on the way it has been reported and discussed. As regards the reporting of *P* values, SPSS-10 and 16 both generate this form, and we have seen such practice in many reputed journals when highly significant *P* values are achieved. Of course there could be different viewpoints. The errors in the discussion section especially those relating to ideas promoted by John Tower[3] are regretted but in no way affect the confidence that we place in our data and its significance.

We continue to stand by our results using *rasayanas* modified to suit *Drosophila* and confirm that they are consistent, significant, and reproducible. However, in the best interest of scientific enquiry and to dispel doubts about our results, we would be happy to work with *Drosophila* experts like Lakhotia. Under suitable nondisclosure agreement, we are also open to sharing required information and data as well as test material for possible repetition of our experiments in other laboratories.

Deeper understanding and appreciation of Ayurveda is essential for the way ahead. It will bring rapid success in these kinds of trans-disciplinary research activity and in collaborations searching for answers to questions concerning concepts from ancient knowledge. We, therefore, feel that it is essential to involve experienced Ayurveda experts on a basis founded on mutual trust and respect for the two seemingly diverse but mutually consistent and compatible disciplines.
